# Supplementary material for: Content-rich biological network constructed by mining PubMed abstracts
Source: BMC Bioinformatics. 2004 Oct 8;5:147. doi: 10.1186/1471-2105-5-147 (PMC528731; doi:10.1186/1471-2105-5-147)
Supplement: Additional File 5 — The original Chilibot query results of the term "long-term potentiation (LTP)" and 22 other terms, limiting the latest references analyzed to the years 1990, 1995, 2000, and 2004. [file 1471-2105-5-147-S5.bz2 › chilibotAdditionalFile5/ltp1995/html/NMDA_AMPA.html]

 


 **NMDA** and **AMPA** 
  
Found 1026 abstracts in PubMed,  **30 abstracts were retrieved and analyzed**.  


---

 Search Google  |
 PDF files only 
|  EDU domain only 

---

**Interactive relationship** (e.g. stimulation, inhibition, etc)

- In striking contrast, pretreatment with a potent and highly selective  **NMDA**  antagonist CGP37849 fully opposed both the  **NMDA**  and the  **AMPA**  mediated elevation of ME mRNA.  Ref: 8869164 Synapse, 1995
- It seems that ACRO exerts its unique pharmacological actions by activating a subclass of non  **NMDA**  receptors distinct from those activated by kainate and  **AMPA** , and may become a useful tool for investigating the biological roles of glutamate receptors.  Ref: 8846097 Brain Res, 1995
- Pretreatment with a potent and highly specific  **AMPA**  antagonist NBQX predictably blocked the  **AMPA**  mediated elevation, and was only slightly effective against the  **NMDA**  induced response.  Ref: 8869164 Synapse, 1995
- **NMDA**  was more effective than  **AMPA**  over the dose range used.  Ref: 8869164 Synapse, 1995
- Interactions between  **NMDA**  and  **AMPA**  kainate receptors in the control of micturition in the rat.  Ref: 8666029 Eur J Pharmacol, 1995
- The compound displaces both  **NMDA**  and  **AMPA**  receptor binding and antagonizes depolarizations in cortical slices evoked by both  **NMDA**  and  **AMPA** .  Ref: 8523401 J Med Chem, 1995
- The rapidly triggered excitotoxicity induced by glutamate was blocked by  **NMDA**  selective antagonists, was calcium dependent and pH sensitive and could be mimicked by  **NMDA**  but not by non  **NMDA**  agonists,  **AMPA** , kainate or quisqualate.  Ref: 8613724 J Neurosci, 1995

**Parallel relationship** (e.g. studied together, co-existance, homology, etc.)

- In the present study, the effects of the fluoroquinolone, ciprofloxacin, alone and in combination with the NSAID, biphenyl acetic acid BPAA, were examined on GABAA,  **NMDA** ,  **AMPA** , and kainate evoked current responses recorded from cultured rat hippocampal neurones, using the whole cell patch clamp technique.  Ref: 8788959 Neuropharmacology, 1995
- These results suggest that both  **AMPA**  kainate and  **NMDA**  glutamate receptors are important in the micturition reflex pathway.  Ref: 8666029 Eur J Pharmacol, 1995
- Three types of glutamate receptor agonists, kainate, quisqualate,  **AMPA**  alpha amino 3 hydroxy 5 methyl 4 isoxazole propionic acid and  **NMDA**  N methyl D aspartate, were effective in inducing the potentiating effect.  Ref: 8788945 J Physiol, 1995
- 2 Me 5HT did not change motoneuron depolarizations generated by addition to the Ringer s solution of the excitatory amino acid agonists  **AMPA**  10 30 microM, kainate 30 microM, or t ACPD 100 microM, but  **NMDA**  induced motoneuron depolarizations 100 microM were significantly and reversibly reduced approximately 20% by exposure to 2 Me 5HT 100 microM.  Ref: 8788913 Brain Res, 1995
- We report the synthesis and characterization of 6 LY246492, which is a competitive N methyl D aspartate  **NMDA**  and 2 amino 3 3 hydroxy 5 methylisoxazol 4 yl propanoic acid  **AMPA**  receptor antagonist.  Ref: 8523401 J Med Chem, 1995
- In situ hybridization histochemistry of forebrain tissue sections from rats 8 h after a single intracerebroventricular infusion of  **NMDA**  or  **AMPA**  revealed a significant and dose dependent elevation to a maximum of almost 50% of striatal ME mRNA when compared to vehicle injected controls.  Ref: 8869164 Synapse, 1995
- The results indicate that activation of kainate but not  **NMDA**  and  **AMPA**  receptors is involved in the indirect, adenosine mediated inhibition by exogenous glutamate of the release of noradrenaline in rabbit brain cortex slices.  Ref: 8750916 Naunyn Schmiedebergs Arch Pharmacol, 1995
- We have tested, in CA1 hippocampal slices, the hypothesis that the expression of long term potentiation LTP by alpha amino 3 hydroxy 5 methyl 4 isoxazolepropionic acid  **AMPA**  and or N methyl D aspartate  **NMDA**  receptors depends on the degree of  **NMDA**  receptors activation during the tetanus.  Ref: 8747197 J Neurophysiol, 1995
- The effects of a range of structurally dissimilar compounds which possess affinity for sigma binding sites were examined on the responses of cultured hippocampal pyramidal neurones to the excitatory amino acid analogues N methyl D aspartate  **NMDA** , kainate and RS alpha amino 3 hydroxy 5 methylisoxazole 4 propionic acid  **AMPA** .  Ref: 8680708 Br J Pharmacol, 1995
- Picrotoxin 250 ng 0.5 microliters injected into the same striatal region 30 min before  **NMDA** ,  **AMPA** , or kainic acid strongly increased the contralateral turning induced by each of those compounds.  Ref: 8787830 Neurosci Lett, 1995
- In mice and pigeons, the compound showed antagonism of responses mediated through  **NMDA**  and  **AMPA**  receptors.  Ref: 8523401 J Med Chem, 1995
- In mouse hippocampal neurones under whole cell voltage clamp, the compounds tested reversibly attenuated  **NMDA** ... evoked currents with a rank order potency IC50 values in microM ifenprodil 0.8 > N allylnormetazocine 1.1 > dextromethorphan 1.8 = haloperidol 1.9 > pentazocine 7.2 > 1S, 2R cis N methyl N 2 dichlorophenyl ethyl 2 1 pyrrolidinyl cyclohexylamine 17 = rimcazole 18 > di 2 tolyl guanidine 37 > opipramol 96 > caramiphen 110 = carbetapentane 112 > > 3 3 hydroxyphenyl N 1 propyl piperidine 485.  Ref: 8680708 Br J Pharmacol, 1995
- In addition, ACEA 1011 inhibited  **AMPA**  preferring non  **NMDA**  receptors by competitive antagonism at glutamate binding sites.  Ref: 8531083 J Pharmacol Exp Ther, 1995
- Further,  **NMDA**  antagonists may not be clinically useful for opiate withdrawal due to their side effects, but  **AMPA**  antagonists may be of great benefit for alleviating opiate withdrawal symptoms in humans.  Ref: 8747753 Neuropsychopharmacology, 1995
- The role of the locus coeruleus and N methyl D aspartic acid  **NMDA**  and  **AMPA**  receptors in opiate withdrawal.  Ref: 8747753 Neuropsychopharmacology, 1995
- 3SR, 4aRS, 6SR, 8aRS 6 1H tetrazol 5 yl decahydroisoquinoline 3 carboxylic acid, a novel, competitive, systemically active  **NMDA**  and  **AMPA**  receptor antagonist.  Ref: 8523401 J Med Chem, 1995
- Expression of LTP by  **AMPA**  and or  **NMDA**  receptors is determined by the extent of  **NMDA**  receptors activation during the tetanus.  Ref: 8747197 J Neurophysiol, 1995
- It was found that long term... ethanol exposure increased levels of immunoreactivity of the NMDAR1 subunit, an obligatory component of  **NMDA**  glutamate receptors, and of the GluR1 subunit, a component of many  **AMPA**  glutamate receptors.  Ref: 8869159 Synapse, 1995
- The inner hair cells IHCs that transduce the mechanical displacements into neural activity, release glutamate on receptor activated channels of  **AMPA** , kainate, and  **NMDA**  types.  Ref: 8787031 Prog Neurobiol, 1995
- 7 NI significantly attenuated lesions produced by intrastriatal injections of  **NMDA** ... and the protection was reversed by L arginine but not by D arginine, 7 NI produced nearly complete protection against striatal lesions produced by systemic administration of 3 nitropropionic acid 3 NP, another succinate dehydrogenase inhibitor, 7 NI protected against malonate induced decreases in ATP, and increases in lactate, as assessed by 1H magnetic resonance spectroscopy.  Ref: 8613773 J Neurosci, 1995
- In unanesthetized decerebrate rats, GYKI 52466 1 4 aminophenyl 4 methyl methylenedioxy 5H benzodiazepine hydrochloride, an  **AMPA**  kainate receptor antagonist, and MK 801 dizocilpine, an  **NMDA**  receptor antagonist, acted synergistically to depress the micturition reflex.  Ref: 8666029 Eur J Pharmacol, 1995
- N Methyl D aspartate  **NMDA** , 500 ng 0.5 mu l, alpha amino 3 hydroxy 5 methyl 4 isoxasole propionic acid  **AMPA** , 1000 ng 0.5 mu l or kainic acid 50 ng 0.5 mu l, injected into the intermediate and caudal parts of the caudate putamen, induced contralateral head turns and rotations.  Ref: 8750734 Eur J Pharmacol, 1995
- These findings indicate that c fos induction in oligodendrocyte progenitors is mediated through the  **AMPA**  kainate receptors, while  **NMDA**  and metabotropic receptor subtypes are not involved.  Ref: 8845939 Eur J Neurosci, 1995
- We attempted to establish the identity of a putative transmitter released by interneurons responsible for the effects on  **NMDA**  depolarizations produced by 2 Me 5HT, but the  **AMPA**  receptor antagonist, CNQX 10 microM, the GABAA receptor antagonist, bicuculline 50 microM, the GABAB receptor antagonist, saclofen 100 microM, the opioid antagonist, naloxone 100 microM, and the adenosine antagonists, CPT 20 100 microM and CSC 10 100 microM did not alter 2 Me 5HT induced reductions of  **NMDA**  depolarizations.  Ref: 8788913 Brain Res, 1995
- Whole cell currents evoked by  **NMDA** ,  **AMPA**  or kainate were little influenced by ciprofloxacin 100 microM, BPAA 100 microM, or ciprofloxacin plus BPAA both at 100 microM.  Ref: 8788959 Neuropharmacology, 1995
- The ranking order was glutamate > kainate >  **NMDA**  >  **AMPA**  > quisqualate.  Ref: 8788945 J Physiol, 1995
- On day 5 of withdrawal, haloperidol decreased the number of contralateral head turns, but did not significantly influence the contralateral rotations induced by  **NMDA** ,  **AMPA**  and kainic acid.  Ref: 8750734 Eur J Pharmacol, 1995
- Noradrenaline 10 50 microM reversibly reduced the amplitude of the pharmacologically isolated excitatory potentials mediated by both  **NMDA**  and alpha amino 3 hydroxy 5 methyl isoxazole propionic acid  **AMPA**  receptors, the former being more strongly affected.  Ref: 8845941 Eur J Neurosci, 1995
- These results suggest that glutamate is released following stimulation of afferent fibers in the ST and acts on both  **AMPA**  kainate and  **NMDA**  glutamate receptors.  Ref: 8846076 Brain Res, 1995
- Neuronal firing rate can be increased by raising the scrotal skin temperature above 36 degrees C, and also by ionophoretic application of either N methyl D aspartate  **NMDA**  or alpha amino 3 hydroxy 5 methyl 4 isoxazole propionic acid  **AMPA**  a non  **NMDA**  receptor agonist.  Ref: 8786854 Neurosci Lett, 1995
- To measure the  **AMPA**  and  **NMDA**  receptor mediated field excitatory postsynaptic potential fEPSPA and fEPSPN, respectively, we have used the following procedure control fEPSPA was first measured, 6 cyano 7 nitroquinoxaline dione CNQX, 10 microM was then added, and fEPSPN was evoked.  Ref: 8747197 J Neurophysiol, 1995
- We investigated the characteristics of the neurotoxicity mediated by non N methyl D aspartic acid  **NMDA**  type glutamate receptors in the spinal cord by infusing rats intrathecally with three specific agonists acromelic acid A ACRO, kainic acid and 1 amino 3 hydroxy 5 methyl 4 isoxazole propionic acid  **AMPA** .  Ref: 8846097 Brain Res, 1995
- We suggest that  **AMPA**  and  **NMDA**  components are potentiated through two different presumably postsynaptic processes.  Ref: 8747197 J Neurophysiol, 1995
- Using the resolved optical isomers of 6, both  **NMDA**  and  **AMPA**  antagonist activities were found to reside in a single isomer, 6.  Ref: 8523401 J Med Chem, 1995
- The present experiment was designed to test the effects of direct activation of the  **NMDA**  or  **AMPA**  types of GLU receptor on the levels of two mRNAs that encode the peptide cotransmitters met5 enkephalin ME and substance P SP.  Ref: 8869164 Synapse, 1995
- N Methyl D aspartate  **NMDA** , 500 ng 0.5 microliters, alpha amino 3 hydroxy 5 methyl 4 isoxasole propionic acid  **AMPA** , 1000 ng 0.5 microliters, or kainic acid 50 ng 0.5 microliters injected into intermediate and caudal parts of the caudate putamen induced contralateral head turns and rotations.  Ref: 8787830 Neurosci Lett, 1995
